# Life-Threatening Coronary Vasospasm Treated by Implantable Cardioverter Defibrillator: The Warning Signs

**DOI:** 10.1155/2022/4504028

**Published:** 2022-07-27

**Authors:** Deborah Adepoju, Jamie A. L. Smith, Stephen J. Leslie

**Affiliations:** Cardiac Unit, Raigmore Hospital, Inverness IV2 3UJ, UK

## Abstract

Coronary artery vasospasm is the sudden narrowing of an artery caused by rapid prolonged contraction. It reduces blood supply to the heart and can present with typical cardiac chest pain symptoms. Vasospasm can lead to fatal arrhythmic complications such as ventricular fibrillation. Our case report describes an example of this occurring in a 53-year-old female, and the management plan that ensued. We look at the importance of accurate and prompt diagnosis of vasospasm and how this can have implications for treatment options. One of the available treatments for vasospasm is placement of an implantable cardioverter defibrillator (ICD). This delivers a shock in the event of future life-threatening arrhythmia, with the aim of preventing cardiac arrest. ICD placement, however, is not always a suitable option. This case report discusses the various challenges that arose while making the decision for ICD placement and gives insight into the best available treatment options for coronary artery vasospasm. We also highlight early warning signs that predict life-threatening vasospastic events and how this can be diagnosed and treated appropriately.

## 1. Learning Objectives

Coronary vasospasm causing chest pain is relatively common. Life-threatening arrhythmias are an important complication of vasospasm. Recurrent episodes of chest pain in the absence of coronary artery disease could be indicative of severe vasospasm. Coronary angiogram and medical therapy should be considered to prevent the onset of vasospasm-induced arrhythmic events. Current literature suggests the best means of preventing death in vasospasm is by combining medical therapy with an implantable cardioverter defibrillator. This decision, however, is not always straight forward and requires careful evaluation, considering all aspects of the clinical case including patient's wishes.

## 2. Introduction

Coronary vasospasm was first described in 1959 as an important cause of myocardial ischaemia [1]. It can manifest in different ways, from asymptomatic ischaemia to sudden cardiac death. Vasospasm is an important cause of life-threatening cardiac arrhythmias in patients without any hemodynamically significant coronary artery disease [1].

Many different factors such as can coronary artery inflammation, oxidative stress, endothelial dysfunction, and lifestyle choices can all increase the likelihood of developing vasospasm. [2]

Although the two often occur together, the diagnosis of vasospasm can often be mislabelled purely as atherosclerotic disease. [3] Accurate diagnosis of vasospasm relies on the use of coronary angiography. When vasospasm leads to a life-threatening arrhythmic event, lifestyle modification is often combined with medical therapy and placement of an implantable cardioverter defibrillator (ICD) to prevent recurrence. The decision to opt for ICD placement is not always simple. Patient factors must be considered, and the benefit-risk assessment must be carefully weighed. An article by Ishihara et al. discussed the pathophysiology behind vasospasm in detail. They further discussed the use of ICD treatment in vasospasm patients postventricular fibrillation- (VF-) induced cardiac arrest and the importance of carefully weighing up the potential benefit of ICD implantation with a reasonable risk stratification beforehand [4].

This report is an example of a classic presentation of spasm-induced cardiac arrest. We discuss some of the difficulties that arose while deciding about ICD placement and how these difficulties were overcome, along with some warning signs that preceded cardiac arrest.

## 3. Case Report

A 53-year-old female chef with a previous history of non ST-elevated myocardial infarction (NSTEMI) and recurrent episodes of chest pain, presented to the Emergency Department after a collapse and confirmed VF arrest. The patient required 2 direct current shocks before return of spontaneous circulation.

Her past medical history includes hypertension, chronic obstructive pulmonary disease (COPD), asthma, and a transient ischemic attack (TIA). She also had a 30 pack-year smoking history and body mass index of 30.3 kg/m^2^. Medications included atorvastatin 40 mg, aspirin 75 mg, and lansoprazole 30 mg.

**Figure 1:**
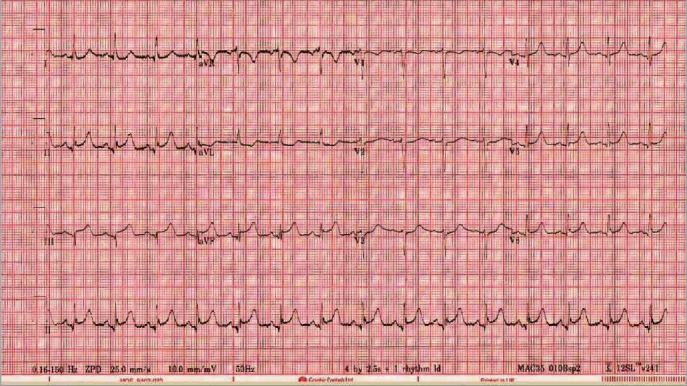
‘Abnormal ECG.' This figure is a 12-lead ECG which was taken after resuscitation from cardiac arrest. It shows ST elevation at the J point in leads II, III, and aVF.

**Figure 2:**
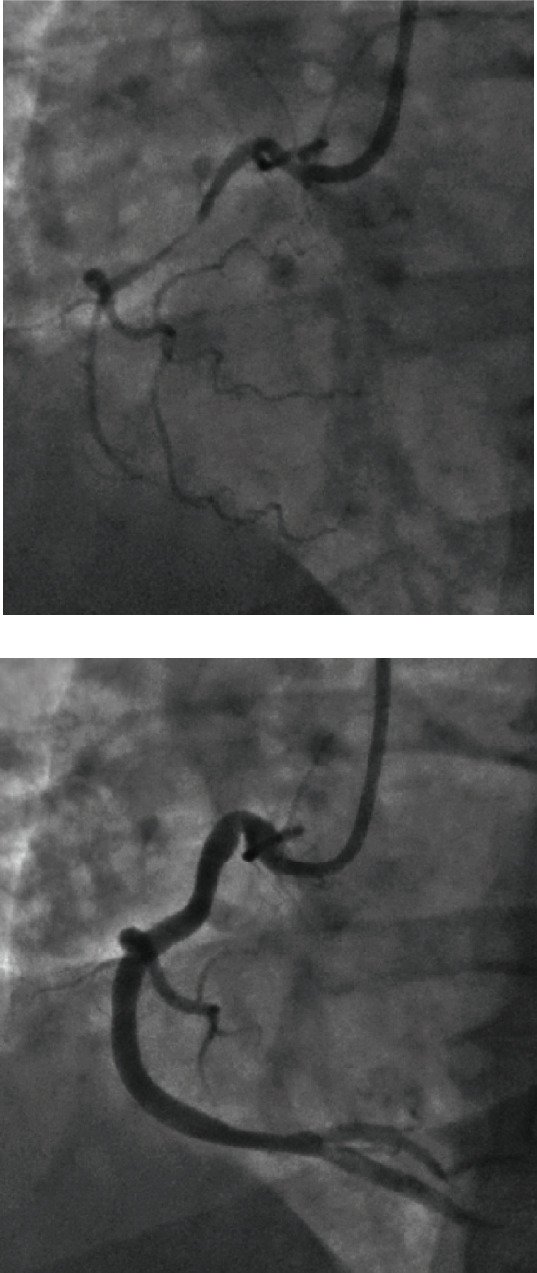
‘Right coronary artery: before and after.' This figure is a coronary angiogram containing 2 images (a, b). (a) Narrowing of the right coronary artery shortly after cardiac arrest. (b) How the narrowing of the same right coronary artery was almost completely restored immediately after administration of 400 micrograms of intracoronary nitrate.

On physical exam, she was sweaty with cool peripheries. Pulse was 113 beats per minute, respiratory rate was 24 breaths per minute, and blood pressure was 86/61 mmHg. A 12-lead ECG revealed ST elevation at the J point in leads II, III, and aVF (Figure 1). Plasma high-sensitivity troponin increased from 27 ng/L to 50 ng/L over four hours. Urea and electrolytes were all within normal limits, and the lipid profile showed cholesterol of 7 mmol/L and triglycerides of 5.7 mmol/L. The clinical diagnosis, supported by ECG, was acute occlusive myocardial infarction, and she was transferred for emergency coronary angiography. ECG had normalized on arrival in the catheterization laboratory; however, coronary angiography showed considerable restriction of the right coronary artery (RCA) lumen (Figure 2(a)), accompanied by recurrent gross ST elevation on the ECG monitor. The patient was given 400 micrograms of intracoronary nitrate. This resulted in a dramatic resolution of the RCA stenosis and ST segments on ECG (Figure 2(b)). Severe vasospasm in the RCA was diagnosed as the primary problem due to the quick resolution of the dynamic ECG changes and near-normal coronary angiogram findings following nitrate administration.

**Figure 3:**
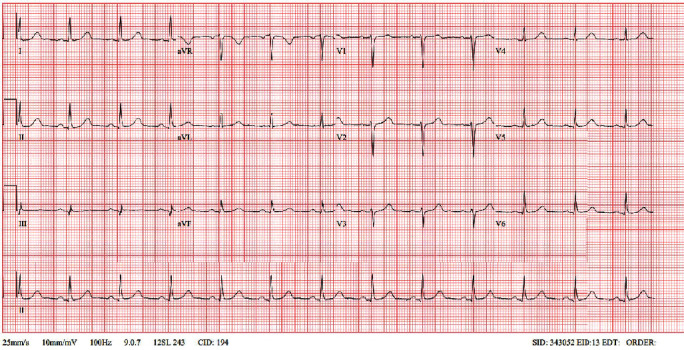
‘Normalized ECG.' This figure is a 12-lead ECG which was taken the day after cardiac arrest. It shows a return to normal sinus rhythm and resolution of the inferior ST-segment elevation.

Following angiography, the patient was started on vasodilator therapy with diltiazem (240 mg) and a long acting nitrate (Elantan 50 mg). There were no recurrences of arrhythmia, and all subsequent resting ECGs showed a return to normal sinus rhythm (Figure 3). An echocardiogram demonstrated preserved LV function. Due to the presentation with a VF arrest, ICD placement was discussed with the patient. This was successfully performed and, coupled with lifestyle modification and medical therapy, she has shown no recurrences of chest pain, ECG changes, or arrhythmias at follow-up.

## 4. Discussion

This patient had multiple risk factors for coronary vasospasm. She was middle aged, a chronic smoker with obesity, hypertension, and high cholesterol. She was facing stress at work which is also a known precipitant for vasospasm [5]. She also suffered from lifelong asthma. Previous studies have shown that asthma is associated with a higher incidence of vasospasm [6]. This concoction of hyperlipidemia, hypertension, smoking, and obesity is ordinarily sufficient to induce severe coronary artery disease. An angiogram done two years before this presentation, however, showed only minor plaque disease. The root cause of her symptoms was therefore most likely due to coronary vasospasm and not atherosclerosis.

The striking feature of this case was the many recurrent episodes of rapidly recovering chest pains in the preceding year. These were never fully investigated, but ECG tracings were normal each time. In retrospect, it is highly likely that these were episodes of spasm. Stern and Bayes de Luna documented a similar case whereby a patient presented with multiple episodes of excruciating chest pain over 2 years and a diagnosis of vasospasm was later confirmed. [7]

Due to the increased risk of life-threatening arrhythmias with vasospasm, there is an additional therapeutic option of ICD placement. In similar cases of spasm-induced cardiac arrest, patients have been managed with a combination of medication and ICD [8]. A previous observational study of patients implanted with ICD following spasm-induced arrests showed no further events in the 5-year follow-up period. [9] Currently, there has not been any randomized trials comparing the combination of ICD and medical therapy to medical therapy alone.

Vasospasm is an important differential in the diagnosis of angina or acute coronary syndrome (ACS) as it is easily treated, and if found early, the onset of life-threatening arrhythmias can be prevented.

An Asian study showed that in patients with stable angina or ACS that was not caused by an obstructive atheroma, vasospasm was the cause in 50% and 57%, respectively [10]. A German study similarly performed intracoronary acetylcholine provocation tests in patients with acute chest pain at rest to establish the prevalence of vasospasm as a cause. In 42 out of 86 tested (49%), coronary vasospasm was confirmed as the source of their symptoms. They thus concluded that vasospasm is a frequent cause of ACS and should more regularly be considered as a differential diagnosis [11].

We suggest that healthcare practitioners more frequently consider that while obstructive atheroma is the most common cause of coronary disease, vasospasm could also be a possibility, as this can have implications in management. Diltiazem is very effective in vasospasm but is contraindicated in treatment of ACS patients with reduced left ventricular ejection fraction. In a vasospasm patient misdiagnosed with ACS, diltiazem could be withheld, and the consequences could be detrimental. In cases where acute chest pain is recurrent despite an absence of plaque disease, vasospasm should be highly suspected, and these patients should have coronary angiogram.

Acute chest pain episodes in a relatively young patient, especially if occurring in the early morning (as was the case with our patient and Stern and Bayes de Luna) [7], are warning signs that should raise the suspicion of coronary vasospasm. This characteristic presentation of pain paired with dynamic ECG changes is a key in diagnosis. Calcium channel blockers coupled with the vasodilatory effect of nitroglycerin are extremely effective in the treatment and prevention of vasospastic complications. ICD therapy is effective in preventing recurrence and can be considered if the patient's vasospasm has led to a life-threatening arrhythmia.
